# Cellular senescence meets infection: highlights from the 10th annual International Cell Senescence Association (ICSA) conference, Rome 2025

**DOI:** 10.18632/aging.206349

**Published:** 2025-12-23

**Authors:** Stefanie Deinhardt-Emmer, François Trottein, Federico Armando, Rocío M. Tolosa, Andrea Rodríguez-Agustín, Victor Casanova, Núria Climent, Pamela Martinez-Orellana, Edoardo Scarpa, Marisa Gariglio, Marco De Andrea

**Affiliations:** 1 Institute of Medical Microbiology, Jena University Hospital, 07747 Jena, Germany; 2 Univ. Lille, CNRS, INSERM, CHU Lille, Institut Pasteur de Lille, U1019 - UMR 9017 - CIIL - Center for Infection and Immunity of Lille, F-59000 Lille, France; 3 Department of Veterinary Medicine, Pathology Unit, University of Parma, 43126 Parma, Italy; 4 Department of Pathology, University of Veterinary Medicine Foundation, 30559 Hannover, Germany; 5 Centro Singular de Investigación en Medicina Molecular y Enfermedades Crónicas (CIMUS), Universidad de Santiago de Compostela, 15782 Santiago de Compostela, A Coruña, Spain; 6 Instituto de Investigación de Santiago de Compostela (IDIS), Universidad de Santiago de Compostela, 15782 Santiago de Compostela, A Coruña, Spain; 7 AIDS and HIV Infection Group, Fundació de Recerca Clínic Barcelona-Institut d’Investigacions Biomédiques August Pi i Sunyer (FRCB-IDIBAPS), 08036 Barcelona, Spain; 8 Universitat de Barcelona (UB), 08036 Barcelona, Spain; 9 Centro de Investigación Biomédica en Red sobre Enfermedades Infecciosas (CIBERINFEC), Instituto de Salud Carlos III (ISCIII), 28029 Madrid, Spain; 10 The International Centre for Genetic Engineering and Biotechnology (ICGEB), 34149 Trieste, Italy; 11 Department of Pharmaceutical Sciences, University of Milan, 20133 Milano, Italy; 12 National Institute of Molecular Genetic (INGM), 20122 Milano, Italy; 13 CAAD - Center for Translational Research on Autoimmune and Allergic Disease, University of Piemonte Orientale, 28100 Novara, Italy; 14 Department of Translational Medicine, University of Piemonte Orientale, 28100 Novara, Italy; 15 Department of Public Health and Pediatric Sciences, University of Turin, Medical School, 10126 Turin, Italy

**Keywords:** infection-driven senescence (IDS), senescence-associated inflammation, host-pathogen interactions, inflammation

## Abstract

At the 10^th^ Annual International Cell Senescence Association (ICSA) Conference, held in Rome (Italy) from September 17–19, 2025, “senescence and infection” emerged as a recurring highlight linking diverse sessions across virology, immunology, and aging research. Presentations addressed virus-induced senescence in influenza A virus (IAV), SARS-CoV-2, cytomegalovirus (CMV), and human immunodeficiency virus (HIV), as well as bacterial infections such as *Mycobacterium abscessus*. Together, these studies have defined infection-driven senescence (IDS) as a critical biological process in both acute disease and long-term sequelae, connecting infectious pathology with mechanisms of aging and chronic inflammation.

Mechanistically, IDS integrates DNA damage responses, inflammatory signaling, and metabolic stress, with consistent activation of p16^INK4a^, p21, and NF-κB pathways. Evidence across immune, epithelial, and neuronal systems have revealed that senescence contributes to impaired regeneration, persistent inflammation, and altered host–pathogen dynamics. Emerging therapeutic data suggest that senolytic or senomorphic strategies may alleviate infection-associated tissue damage.

Collectively, the conference highlighted IDS as an expanding frontier that bridges infection biology and aging research, emphasizing its potential relevance for prevention and therapy of chronic age-related disease.

## INTRODUCTION: SENESCENCE AT THE CROSSROADS OF AGING AND INFECTION

Cellular senescence is a fundamental biological process, originally characterized as an irreversible cell cycle arrest that limits proliferation of damaged or aged cells [1]. Over the past three decades—largely due to the pioneering work of Judith Campisi and colleagues—senescence has been redefined as a multifaceted stress response with both protective and deleterious consequences [2, 3]. While senescent cells act as barriers against malignant transformation and play roles in tissue remodeling and repair, their persistence and pro-inflammatory secretome—known as the senescence-associated secretory phenotype (SASP)—fuel chronic inflammation, tissue degeneration, and aging-associated pathologies [4–6].

Traditionally studied in the contexts of cancer and aging, senescence has only recently entered the spotlight in infection biology. A growing body of evidence suggests that microbes can trigger senescence directly through cellular damage or indirectly through paracrine signaling. Infections may thus induce senescence as part of an antiviral or antibacterial defense program, while pathogens in turn evolve strategies to exploit, subvert, or persist within senescent environments. The outcome is a complex and sometimes paradoxical interplay: senescence can restrict pathogen replication but can also provide niches for persistence and drive long-term tissue dysfunction.

At the 2025 International Cell Senescence Association (ICSA) meeting held in Rome, Italy, IDS emerged as a unifying theme across the talks presented in the session on infections and senescence, which was sponsored by the Department of Excellence in Aging, University of Piemonte Orientale, Novara - Italy. Presentations spanned virus-induced senescence (VIS) from influenza, SARS-CoV-2, CMV, and HIV to bacterial infections such as *Mycobacterium abscessus*.

Here we will synthesize these highlights, outline common mechanisms, and discuss therapeutic opportunities, framing IDS as an emerging frontier in medicine—one that echoes and extends Judith Campisi’s enduring scientific legacy.

## Infections and senescence: from acute pathology to long-term sequelae

Respiratory viral infections remain among the most significant global health threats, particularly in older adults. François Trottein’s (Institut Pasteur de Lille, France) research team investigates the role of age-related senescent cells in respiratory infections. Using influenza and COVID-19 models in aged animals, they showed that pharmacologic depletion of senescent cells with navitoclax, a Bcl-2 family inhibitor, reduces acute pulmonary inflammation and mitigates long-term pulmonary sequelae [7] and submitted]. In SARS-CoV-2 infection, prophylactic clearance of senescent cells decreases viral replication, likely due to their high expression of the viral receptor ACE2. In young mice, both pharmacologic and genetic removal of senescent cells (p16-ATTAC mice) also reduces post-influenza pulmonary sequelae [8]. Importantly, they observed that senescent cell depletion accelerates epithelial repair even after virus clearance, suggesting a therapeutic window beyond the acute phase. Ongoing work of this group focuses on elucidating the molecular pathways by which senescent cell clearance confers protection with the aim of investigating the translational potential of senolytic therapies in viral pneumonia.

Stefanie Deinhardt-Emmer (Jena University Hospital, Germany) presented compelling evidence that respiratory viruses themselves can drive premature cellular senescence. She showed that Influenza A virus (IAV) triggers an indirect senescence program via TNF-dependent macrophage activation, whereas the SARS-CoV-2 omicron variant directly induces senescence in infected lung epithelial cells [9, 10]. Hallmarks include p16^INK4a^, p21, and p38 activation alongside a robust SASP. Importantly, senescent fibroblasts in infected tissue exhibited increased susceptibility to secondary infections, coupled with altered intracellular pH, JAK/STAT activation, and TRAIL expression. These findings position VIS as a central mechanism not only in acute pathology but also in shaping tissue environments that perpetuate infection and inflammation [11].

The long-term consequences of respiratory viral infections were further explored by Federico Armando (University of Parma, Italy; University of Hannover, Germany), who examined alveolar progenitors in a hamster model of SARS-CoV-2 infection. In this setting, alveolar differentiation intermediate (ADI) cells displayed atypical morphology, p53 expression, and transcriptomic features consistent with VIS [12]. While these senescent cells declined after 28 days, airway progenitors replaced them, potentially explaining chronic remodeling and the impaired gas exchange seen in long-COVID. These data suggest that IDS in progenitor populations may act as a gateway to persistent lung dysfunction, as supported by *in vivo* lung measurements up to 4 months after infection of this study [13].

Together, these studies underscore that respiratory viruses exploit and induce senescence at multiple levels—through immunosenescence, direct infection-driven programs, and paracrine mechanisms—creating a pro-inflammatory, permissive environment that extends pathology well beyond the acute phase.

Human cytomegalovirus (CMV), a ubiquitous β-herpesvirus, is a paradigm of chronic infection and persistence. In immunocompromised patients and transplant recipients, CMV reactivation can lead to acute severe disease. In addition to that, CMV infection is increasingly linked to aging-related comorbidities, including fatigue syndrome and vascular dysfunctions. In collaboration with Marisa Gariglio (University of Piemonte Orientale, Italy), Marco De Andrea (University of Turin and University of Piemonte Orientale, Italy) previously showed that CMV triggers senescence and paracrine inflammation in renal proximal tubular epithelial cells [14]. At the 2025 ICSA meeting, they reported that similar mechanisms can also occur in human endothelial cells—a key cellular target of CMV *in vivo*. Infected human umbilical vein endothelial cells (HUVECs) displayed multiple hallmarks of senescence, including reduced EdU incorporation, increased p16^INK4a^ expression, and loss of Ki67, with most Ki67-negative cells accumulating in G_0_, consistent with deep and stable cell cycle exit. SASP-associated cytokines were also significantly increased from 4 days post-infection onward, especially IL-6, IL-8, CCL5, IP-10, and IFN-β. Transcriptomic profiling confirmed enrichment of the SenMayo gene set [15], reinforcing activation of a canonical senescence program. They claimed that VIS was not limited to infected cells: through paracrine signaling, inflammation spread to neighboring uninfected cells, as shown by enhanced NF-κB nuclear translocation in both compartments. In summary, the highly inflammatory phenotype of infected endothelial cells—and its transmission to bystanders—may partially explain some of the degenerative complications often observed in patients with prolonged CMV viremia, including vascular disorders.

Complementary data presented by Rocío M. Tolosa from the Carmen Rivas laboratory (University of Santiago de Compostela, Spain), in collaboration with the Manuel Collado group, confirmed that CMV infection elicits DNA damage, p53 activation, and elevated senescence-associated β-galactosidase activity. Consistent with earlier findings from this group [16], senescent cells were markedly less permissive to CMV replication, supporting the view that senescence acts as an intrinsic antiviral defense. These authors further proposed that the accumulation of senescent cells may contribute to vascular inflammation and tissue dysfunction, highlighting the dual role of senescence in CMV pathobiology.

Despite the success of antiretroviral therapy (ART), people with HIV (PWH) exhibit premature aging and increased rates of age-related comorbidities. Multiple presentations at ICSA 2025 highlighted senescence as a key mechanism in this setting. Andrea Rodríguez-Agustín and Víctor Casanova (FRCB-IDIBAPS, Barcelona, Spain) showed that the HIV-1 Tat protein induces DNA methylation changes, acting as an epigenetic driver of the accelerated aging phenotype observed in PWH [17]. Previously, other authors reported that HIV-1 infection reshapes the host DNA methylation landscape in PWH, which is not fully restored by ART [18]. Tat expression led to transcriptional reprogramming favoring inflammation, lipid metabolism alterations, and anti-apoptotic processes [19]. Indeed, it has been described that full-length Tat protein can directly modulate the transcription of more than 400 genes in CD4^+^ T cells [20]*.* Moreover, they demonstrated that intracellular Tat upregulates senescence markers in CD4^+^ T cells, including p21^CIP1^, p16^INK4a^, and γH2AX, along with secretion of SASP mediators [21]. These findings are consistent with observations reported by other authors in which Tat induces cellular senescence in microglia [22] and in primary CD4^+^T models [23], where this protein increases BCL-2 expression, induces apoptosis resistance mediated by FasL and ROS generation. These findings extend the senescence paradigm beyond bystander cell types to the primary targets of HIV infection.

Finally, Núria Climent (CIBERINFEC, FRCB-IDIBAPS, Barcelona, Spain) reported that PWH with or without ART had increased cellular senescence biomarkers in T cells (SA-β-gal, p16^INK4a^, γH2AX, and BCL-2) and SASP in plasma (including IL-6, IL-8, IL-10, RANTES, and CXCL1), especially at chronic and advanced stages of HIV infection. Those results are consistent with previous studies where p16^INK4a^ in PWH T cells and inflammatory cytokines in plasma were higher [24, 25]. PWH also had increased soluble exhaustion biomarkers in plasma such as PD-1, PD-L1, PD-L2, LAG-3, CTLA-4, and TIM-3, even under ART, consistent with reports from other authors [26]*.* In addition, *ex vivo* treatment with dasatinib plus quercetin (D+Q), a senolytic combination currently used in humans [27], induced senolytic activity and reduced senescence biomarkers in PBMCs from PWH with or without ART, including SA-β-gal, p16^INK4a^, and 24 key SASP cytokines. This proof-of-principle suggests that senotherapeutics could mitigate inflammaging in HIV, even under ART.

Collectively, these studies highlight how a persistent viral protein can rewire host epigenomes and senescence programs, providing mechanistic insight into the phenomenon of HIV-associated accelerated aging, while pointing toward senolytics such as D+Q as potential interventions to counteract cellular senescence.

Neurological sequelae of viral infections are increasingly linked to glial cells activation. Pamela Martinez-Orellana (ICGEB, Trieste, Italy) showed that SARS-CoV-2 and tick-borne encephalitis virus (TBEV) can induce DNA damage and senescence in neurons and glial cells. In a previous work, they demonstrated that SARS-CoV-2 infection degrades CHK1 and RRM2, leading to nucleotide pool depletion, S-phase stress, DNA damage accumulation and cellular senescence *in vitro*, *in vivo* and in COVID-19 patients [28]. Activated glial cells are key players in the response to central nervous system infection, yet they are also implicated in inflammation and neurodegeneration. They showed that after SARS-CoV-2 or TBEV infection, human astrocytes and microglia display signs of senescence (increase of p21^CIP1^ and SA-β-gal), activation of cGAS-STING pathway drove by the pro-inflammatory cytokine release (CCL2, CCL5, TNF and IFN-β), and virus induced DNA damage, as observed by the increment of γH2AX positive cells. In rat cortical cultures, effective SARS-CoV-2 infection of the glia led to a major loss of synaptic connections, an increase of cGAS-STING pro-inflammatory response, and an increment of DNA damage foci. In addition, by using an antagonist of the cGAS–STING pathway, they were able to rescue the post-infection decrease in electrical activity. [29]. Taken together, their results suggest that human glial cells are key to initiating the inflammatory cascade in the brain upon viral infection, thereby contributing to long-lasting neuropathology.

Senescence is not restricted to viral infections. Edoardo Scarpa (University of Milan, Italy) presented compelling evidence that *Mycobacterium abscessus* (Mab) causes cellular senescence in macrophages both *in vitro* and in mouse models. In murine alveolar-like macrophages (MPI cells) infected for 5 days, transcriptomic profiling revealed extensive reprogramming during chronic infection, with coordinated upregulation of senescence gene signatures. Likewise, chronically infected cells demonstrated canonical senescence hallmarks, including differential expression of genes related to cellular senescence, elevated p21^CIP1^ expression, SA-β-gal activity, DNA damage response activation (γH2AX and 53BP1 foci), and SASP production and release. Notably, they found that senescence from infected cells resulted transmissible to neighboring uninfected cells via paracrine SASP factors [30]. This paracrine transmission also increased susceptibility to subsequent Mab infection, suggesting amplification of the infected niche and a potential mechanism of infection spreading. Most importantly, senolytic therapy using Navitoclax (a BCL-2 family inhibitor) [31] selectively eliminated senescent macrophages and significantly reduced the bacterial burden. Finally, they also demonstrated that signatures of cellular senescence were present in the lungs of chronically infected mice, and that their treatment with Navitoclax also reduced bacterial burden.

Overall, their findings suggest that chronic bacterial pathogens exploit senescent cellular niches for persistence, and that senolytics could represent promising adjunctive host-directed therapies in mycobacterial disease. This work broadens the paradigm of infection-driven senescence (IDS) beyond viruses and emphasizes its generality as a host response to persistent intracellular pathogens, with therapeutic implications for chronic infections.

## CONCLUSIONS AND TRIBUTE

Here we propose the concept of infection-driven senescence (IDS) to describe the phenomenon in which microbial agents, beyond viruses, can trigger cellular senescence in host cells. While pathogens are clear candidates, whether commensal organisms elicit similar responses remains unresolved and deserves further investigations. Senescence is not merely a hallmark of aging—it is a dynamic cellular program that intersects with infection biology, immunity, and chronic disease. Findings from ICSA 2025 highlight IDS as both a barrier and a liability: it can restrict pathogen replication, yet it fuels chronic inflammation, immunosenescence, and long-term sequelae. Pathogens from influenza and SARS-CoV-2 to CMV, HIV, and mycobacteria engage senescence as part of their host interactions, leaving imprints that extend into aging trajectories.

Therapeutic modulation of senescence—whether through clearance, reprogramming, or suppression of the SASP—represents a new frontier in infectious disease management. As the field advances, it will continue to draw inspiration from Judith Campisi’s pioneering insights into the complexity of senescence and its context-dependent roles.

In honoring her contributions, we also chart a path forward: integrating infection biology with aging research to better understand and ultimately mitigate the shared burden of senescence in human health.
